# The Medical Bill

**Published:** 1883-07

**Authors:** 


### THE MEDICAL BILL.

We see from the proceedings of the Legislature that this bill has been indefinitely postponed. We do not know by whose motion this was done, which is equivalent to the definite, cold-blooded killing of the measure. Could it have been a pea-green doctor, as is too often the case—one of those pill-rollers of a dark corner, whose prescriptions, when drunk (that is, the pill roller), his constituents say they would reether trust than all the docterin of all the other doctors in the world? Be it so.

An argument that proves too much proves nothing. A law that tramples upon common sense and the public interest must, in the nature of the case, be forever inoperative. It may remain a felony in the scope of the law to snatch a dead body for scientific purposes, but dead bodies will continue to be scientifically snatched all the same.

